# Discovering patterns in outpatient neurology appointments using state sequence analysis

**DOI:** 10.1186/s12913-023-10218-y

**Published:** 2023-11-06

**Authors:** Fran Biggin, Quinta Ashcroft, Timothy Howcroft, Jo Knight, Hedley Emsley

**Affiliations:** 1 Lancaster Medical School, Lancaster University, Bailrigg, Lancaster, LA1 4YG UK; 2 Lancashire Teaching Hospitals NHS Foundation Trust, Royal Preston Hospital, Sharoe Green Lane, Fulwood, Preston, PR2 9HT UK; 3 Department of Neurology, Lancashire Teaching Hospitals NHS Foundation Trust, Royal Preston Hospital, Sharoe Green Lane, Fulwood, Preston, PR2 9HT UK

**Keywords:** State sequence analysis, Outpatient appointments, Neurology, Routinely collected data

## Abstract

**Background:**

Outpatient services in the UK, and in particular outpatient neurology services, are under considerable pressure with an ever-increasing gap between capacity and demand. To improve services, we first need to understand the current situation. This study aims to explore the patterns of appointment type seen in outpatient neurology, in order to identify potential opportunities for change.

**Methods:**

We use State Sequence Analysis (SSA) on routinely collected data from a single neurology outpatient clinic. SSA is an exploratory methodology which allows patterns within sequences of appointments to be discovered. We analyse sequences of appointments for the 18 months following a new appointment. Using SSA we create groups of similar appointment sequence patterns, and then analyse these clusters to determine if there are particular sequences common to different diagnostic categories.

**Results:**

Of 1315 patients 887 patients had only one appointment. Among the 428 patients who had more than one appointment a 6 monthly cycle of appointments was apparent. SSA revealed that there were 11 distinct clusters of appointment sequence patterns. Further analysis showed that there are 3 diagnosis categories which have significant influence over which cluster a patient falls into: seizure/epilepsy, movement disorders, and headache.

**Conclusions:**

Neurology outpatient appointment sequences show great diversity, but there are some patterns which are common to specific diagnostic categories. Information about these common patterns could be used to inform the structure of future outpatient appointments.

**Supplementary Information:**

The online version contains supplementary material available at 10.1186/s12913-023-10218-y.

## Background

Outpatient care in the UK is under considerable pressure [1], and in response to this the NHS (National Health Service) has initiated a programme for strategic transformation. The NHS Outpatient Recovery and Transformation programme aims to ‘deliver a personalised outpatient model that better meets individual patient need and improves quality of care and patient outcomes’ [2]. The current model of outpatient care delivery is based on a traditional standard that has not been subject to significant scrutiny or quantitative analysis. In order to determine the nature of any change required, we need to understand the current situation, including current outpatient resource utilisation. The aim of any future recommended changes is to ensure optimal use of available resources, releasing capacity where possible, and improving access to care.

Neurology services in the UK are under particular pressure [3, 4], with a large gap between capacity and demand, and this is especially severe in the geographical area covered in this study (Lancashire and South Cumbria) [5]. The majority of neurology care in the UK is provided in an outpatient setting, so with the current drive for improvements in outpatient care in general and the capacity gap for neurology in particular, there is a pressing need to understand the pressures and potential opportunities for change in this specialty. Although this study is focused on a neurology clinic in England, similar pressures are being experienced elsewhere, and the principles this study is based on are transferable to other geographical and clinical areas.

Understanding the nature of outpatient resource utilisation such as the type of appointments that patients attend, and the order and frequency with which they occur, is useful for both resource planning and improving patient access to appropriate care. An analytical technique called State Sequence Analysis (SSA) has been used in other fields, in particular social sciences, to study patterns in longitudinal data [6, 7]. SSA is used to identify groups of common patterns or sequences of ‘states’ that occur over time. It is a relatively new methodology to healthcare, but a number of studies in the last few years have used SSA. These studies fall into two primary types; those which examine temporal data such as patterns of drug adherence [8, 9] or mortality following illness [10]; and others which study trajectories of care (for example appointment sequences and hospital stays) which are examined in the following paragraph.

Examples of studies which have investigated care trajectory or patient pathway include Le Meur et al. who used SSA to study care consumption in pre-natal care [11] and to examine the determinants of care trajectories in end-stage renal disease [12]. Vanasse et al. used the technique to study healthcare use after hospitalisation with Chronic Obstructive Pulmonary Disease [13]. The same team also used SSA to study care trajectories preceding a diagnosis of schizophrenia [14]. Other recent studies using SSA in healthcare include an examination of social inequalities in care trajectories following a diagnosis of diabetes [15], and a study of referral trajectories in patients with vertigo [16].

Some studies have applied SSA to neurology. For example, in 2021 LeBlanc et al. used SSA in their study of disease modifying therapy (DMT) usage in patients with multiple sclerosis (MS) [17]. They used SSA to identify patterns of DMT use and were able to classify patients into groups with similar usage patterns. In addition, Roux et al. used SSA to analyse care pathways of patients with MS [18, 19]. In their 2019 study they analysed the amount of care that patients ‘consumed’, including GP (General Practitioner) consultations, consultations with a neurologist, and hospital admissions. They were able to identify five different groups of patients with distinct levels of care consumption. In their 2021 study they compared groups of patients with incident and prevalent MS and extended their methodology to include ‘multiple channels’. In this study they use multi-channel SSA to identify 12 care consumption groups for patients with incident MS and 6 groups for prevalent MS.

These previous studies show that it is possible to use SSA in a healthcare setting – including within neurology – to group patients by differing levels of care consumption, drug adherence, and patterns of care observed over time. Previous studies in neurology using SSA have only analysed a single diagnosis in multiple settings, in this study we analyse the number, type and order of appointments across all diagnoses in a single neurology outpatient clinic. We aim to discover common patterns in types of appointment, the number of appointments attended, and the interval at which appointments occur. We will use SSA to create groups of similar appointment sequences and then analyse these groups to determine if there are particular sequences common to different diagnostic categories.

## Methods

### The study population, design and variables

This is a retrospective observational study using SSA to explore patterns in patient appointments in the 18 months following a new appointment. We used routinely collected data from neurology outpatient appointments, from a single clinician, collected over a period of approximately three years and four months (18^th^ September 2015 to 9^th^ January 2019). Data were drawn both from those recorded by the clinician at the time of the appointment, and from administrative information collected by the hospital business intelligence team.

The variables used to create the sequences include the date of an appointment, whether the appointment was attended, if a test was ordered from an appointment and whether a patient was discharged following an appointment. A number of variables were used in further analysis after the sequences had been constructed and clustered, including the diagnosis given to a patient, the patient’s age at the first appointment, the sex of the patient, and the time from referral to the patient’s first appointment.

### SSA methodology

#### Identifying timeframes, defining states and building sequences

**Fig. 1:**
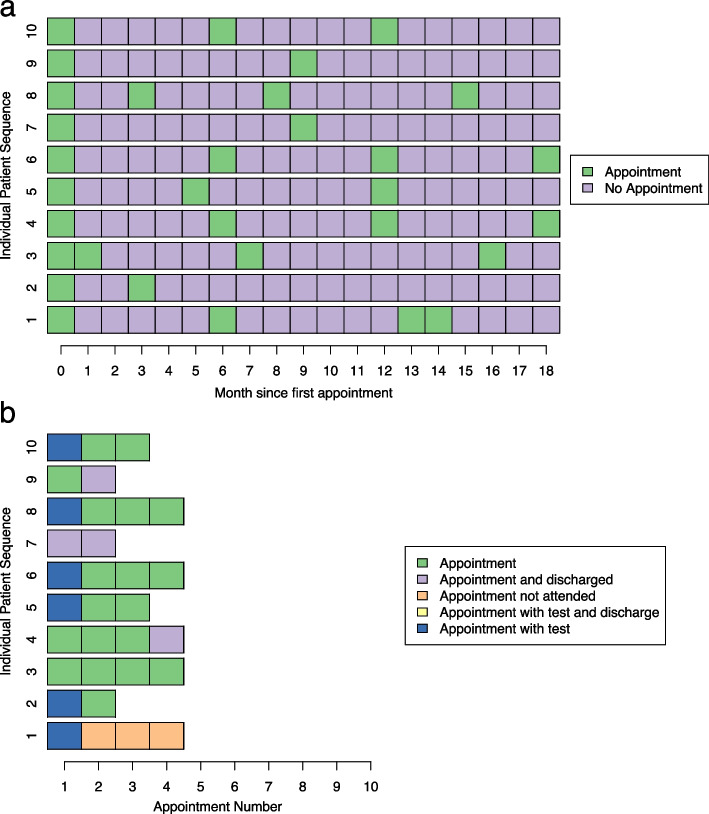
Example of the two types of sequence. **a** Timing of appointments within the 18-month period and **b** Appointment type

To analyse both the timing of appointments and the patterns found in types of appointments we created two separate sets of sequences from the same data. First, we created a set of sequences showing whether an appointment took place in a certain month; this can be seen in Fig. 1a. In this sequence set we used two simple states of “Appointment” and “No Appointment”.

The second set of sequences included only the months in which an appointment was recorded but incorporated additional information about the type of appointment that occurred (Fig. 1b). In this sequence set five mutually exclusive states were defined as: an attended appointment without either a recorded test, or discharge (A); an appointment where a test was ordered (AwT); an appointment at which a patient was discharged (AD); an appointment where a test was ordered and the patient was discharged (ATD); and an appointment that was unattended (ANA). Unattended appointments included cancellations by both the clinic and the patient, and ‘did not attends’ i.e., where a patient did not cancel, but did not turn up at their allotted time. Organising the data into two different types of sequence allowed for separate analysis of different aspects of patient appointment patterns.

#### Measuring dissimilarity between sequences

We used Optimal Matching and Hamming distance algorithms to measure dissimilarity numerically between sequences and create the matrices required for clustering. For the sequences based on the timing of appointments we used Hamming distance as this is the most common method applied to sequences of the same length. In addition Hamming distance does not use insertion and deletion and so it preserves the order of the states and the timing of the appointments [20]. Optimal Matching allows for sequences of differing lengths to be compared and was used for the sequences of appointment types [21].

Both algorithms rely on the principle of assigning a value to the number of operations required to turn one sequence into the other. For example, the sequence A-A-A can be transformed into sequence A-B-A by replacing the middle character with a B. We can assign a numerical ‘cost’ to this operation, for example a value of 1 and then compare costs of transformation between all different sequences.

#### Clustering

We used hierarchical agglomerative clustering with Ward’s criterion [22]. This type of clustering assumes that every individual data point initially belongs in its own cluster, these clusters are compared, and the most similar data points are joined to form clusters. The algorithm then compares these new clusters and again joins the most similar together, and so on until there is only one large cluster with all the data points contained within it. Once the clustering is complete it is necessary to determine the optimal number of clusters.

#### Optimising the number of clusters

The optimal number was chosen using average silhouette width [23]. Silhouette width measures how similar a sequence is to the cluster to which it has been assigned and compares this to how different it is from the other clusters. Average silhouette width is the average of the silhouette width of all the individual sequences and thus measures how well defined (on average) the clusters are, as well as whether each individual sequence has been placed in the ‘correct’ cluster. The metric ranges in value from -1 to 1 with -1 indicating that the clusters are not well defined and individual sequences are not likely to be placed in the ‘correct’ cluster. A score of 1 indicates that the clusters are perfectly separated, and each sequence is very likely to be assigned to the ‘correct’ cluster.

### Hypothesis testing

After selecting the optimal number of clusters we extracted the diagnosis and demographic information for the patients falling into each cluster. Using chi squared tests and t-tests (where appropriate) we were able to determine if cluster membership was independent from these demographic factors. Analysis included diagnosis category, age at first appointment, sex, and time from referral to first appointment.

### Ethics

The research proposal underwent ethical review with both the NHS Research Ethics Committee (Ref: 19/NW/0178) and Confidentiality Advisory Group (Ref: 19/CAG/0056) and received approval from the Health Research Authority (HRA) on 30 May 2019 (Ref: 255676). In addition, the study underwent ethical review with Lancaster University Faculty of Health and Medicine Research Ethics Committee and obtained approval on 17 June 2019 (Ref: FHMREC18092).

## Results

### Selection criteria

**Fig. 2:**
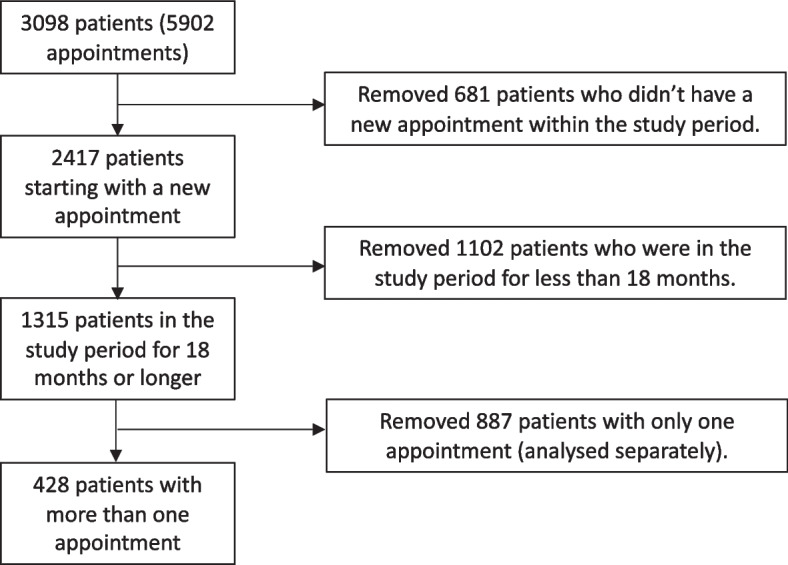
Flow chart showing selection criteria for the study

During the study period data was recorded from 3098 patients who, between them, had 5902 appointments. As patients entered and left the study period at different times, only patients who had a first appointment at least 18 months before the end of the study period were included (see Fig. 2). In addition, many patients only attended one appointment and these patients were removed for separate analysis. This left 428 patients to be included in the sequences analysis.

### Patient characteristics

**Fig. 3:**
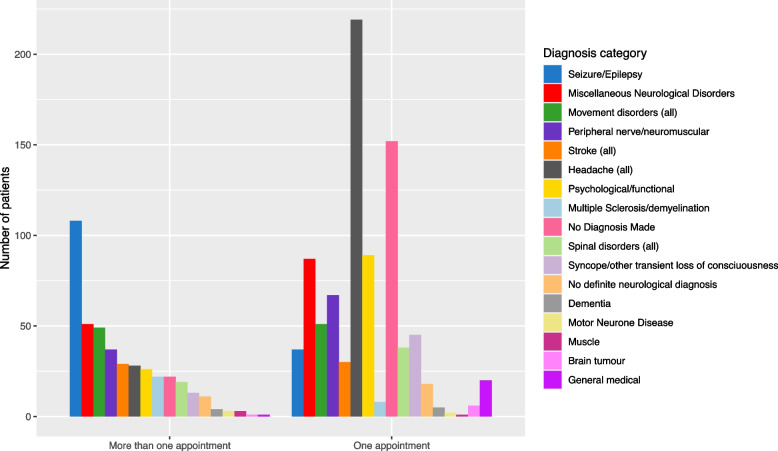
The number of patients within each diagnostic category who return for more than one appointment, compared to those who only have one appointment

Of the 1315 patients who had new appointments followed by at least 18 months of data in the study period, 887 only had one appointment. Table 1 shows the baseline characteristics for these 1315 patients, split to allow comparison between those with only one appointment to those with sequences of two or more appointments. Figure 3 displays the numbers of patients falling into each diagnostic category, directly comparing those who return for more than one appointment with those who only have one appointment.

**Table 1:** Patient and appointment characteristics at the first appointment

	Number with one appointment. Total = 887	Number with more than one appointment Total = 428
Sex (%):		
Female	504 (57)	210 (49)
Male	373 (43)	217 (51)
Mean age at first appointment (SD)	49.6 (18.9)	49.2 (18.7)
Time from referral in weeks (SD)	14.6 (9.6)	13.0 (8.9)
Diagnosis Category (%):		
Seizure/epilepsy	37 (4.2)	109 (25.5)
Miscellaneous Neurological Disorders	87 (9.9)	51 (11.9)
Movement Disorders	51 (5.8)	49 (11.4)
Peripheral nerve/neuromuscular	67 (7.6)	37 (8.6)
Stroke	30 (3.4)	29 (6.8)
Headache	219 (25.0)	28 (6.5)
Psychological/functional	89 (10.1)	25 (5.8)
Multiple Sclerosis/demyelination	8 (0.1)	22 (5.1)
No Diagnosis Made	152 (17.3)	22 (5.1)
Spinal disorders	38 (4.3)	19 (4.4)
Syncope/transient loss of consciousness	45 (5.1)	12 (3.0)
No definite neurological diagnosis	18 (2.1)	11 (2.5)
Dementia	5 (0.1)	4 (0.9)
Muscle	1 (0.01)	3 (0.7)
Motor Neurone Disease	2 (0.02)	3 (0.7)
Brain tumour	6 (0.1)	1 (0.01)
General medical	22 (2.3)	1 (0.01)
Appointment Type (%):		
Appointment and discharge	381 (43.4)	16 (3.7)
Appointment, test request and discharge	314 (35.8)	45 (10.5)
Appointment not Attended	140 (16.0)	17 (3.9)
Appointment	22 (2.5)	134 (31.2)
Appointment with test request	20 (2.3)	216 (50.4)

The mean age at first appointment is similar for both groups of patients – 49.6 for those who only have one appointment compared to 49.2 for those who attend more than one appointment. The time from referral is also similar, 14.6 vs 13 weeks.

In Fig. 3 we see that, overall, the greatest number of patients attend only one appointment. We also see marked differences in the numbers of patients in each diagnostic category. When referring to both Fig. 3 and Table 1 we see that headache (25.0%) and psychological/functional (10.1%) were the most frequent diagnostic categories seen in patients with only one appointment. In addition, patients with only one appointment have a large proportion of unattended appointments (16%) which leads to a high rate of patients where no diagnosis was made (17.3%). Within the group of patients who go on to have more than one appointment, the most common diagnosis is seizure/epilepsy (25.5%), followed by movement disorders (11.4%). The rate of unattended first appointments is much lower in this group (3.9%).

### Timing of appointments

**Fig. 4:**
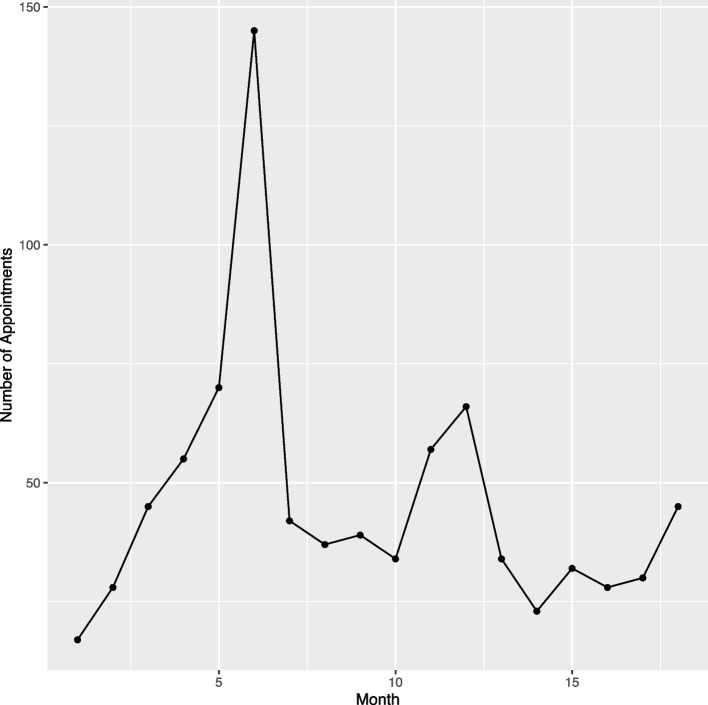
Number of follow-up appointments each month

Within the group of patients with more than one appointment there is a predominant underlying 6 monthly cycle of appointments, as seen in Fig. 4. After a first appointment (at month zero) most patients return at, or around, the 6 month mark. There is then another peak around 12 months, and a smaller peak at 18 months. Very few patients return in the first or second month following their first appointment, and there is a general decline in the number of appointments after 6 months.

In order to investigate the timing of appointments more thoroughly we carried out SSA on sequences with two simple states (see Fig. 1 in the methods section for a visual representation of these types of sequences). We found that the optimal number of clusters was five and descriptions of these groups can be found in Table 2. The largest cluster represents patients who return after 6 months for a second appointment. Further analysis of the patients belonging to each of these clusters revealed few other insights. The only significant result being that patients with movement disorders tended to fall more predominantly into cluster 2, with a second appointment at 3 months followed by further follow up at 6 month intervals. Visualisations of these clusters and a table of patient characteristics for each cluster can be found in Additional file 1.

**Table 2:** Description of the clusters based on SSA of sequences focused on appointment timing

Cluster	Description
1 (*n* = 163)	Patients return for a second appointment after six months
2 (*n* = 49)	Patients return for a second appointment after three months and a third appointment after a further six months
3 (*n* = 89)	Patients return for a second or third appointment at seven or eight months
4 (*n* = 71)	Patients return for a second appointment after five months
5 (*n* = 54)	Patients return for a second appointment after four months and a third appointment at eleven months

### Number and type of appointment

**Fig. 5:**
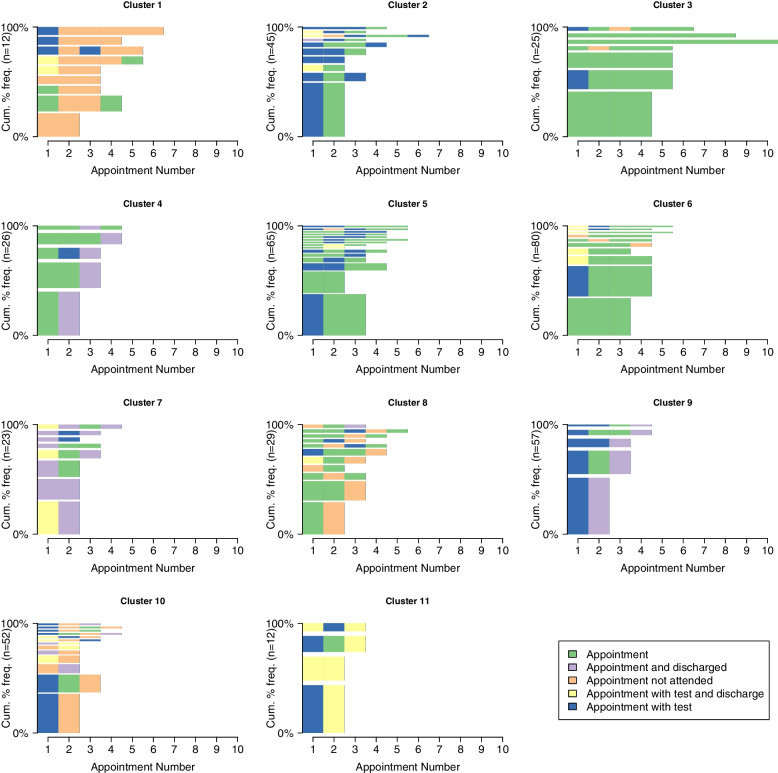
Visualisation of the sequences belonging to each of the 11 distinct clusters. The most common sequence in each cluster is oriented at the base of the y-axis and the height of the bars represents the frequency of that sequence within the cluster. Cluster size is included in brackets in the y-axis title

State Sequence Analysis of the second set of sequences, those with different appointment types, revealed an optimal cluster solution of 11 distinct clusters (see Fig. 5), these clusters are described in Table 3. The largest cluster is cluster 6 which represents patients with three or more appointments within the 18 month period, mainly of appointments without tests or discharges. It is interesting to note that there are a number of patients who are discharged on their first appointment, yet still return for further appointments during the following 18 months, as seen in cluster 7 for example.

**Table 3:** Description of the clusters based on SSA of sequences focused on appointment type

Cluster	Description
1 (*n* = 12)	Two or more unattended appointments in a row
2 (*n* = 45)	An initial appointment with a test followed by a second standard appointment
3 (*n* = 25)	Longer sequences of mainly standard appointments
4 (*n* = 26)	One to three standard appointments, followed by a discharge
5 (*n* = 65)	Two standard appointments in a row, with some appointments with a test
6 (*n* = 80)	Three standard appointments in a row
7 (*n* = 23)	First appointment is a discharge, or a test with a discharge, and the final appointment is also a discharge
8 (*n* = 29)	End with an unattended appointment
9 (*n* = 57)	Initial appointment with a test followed by a discharge
10 (*n* = 52)	Initial appointment with a test followed by an unattended appointment
11 (*n* = 12)	Initial appointment with a test followed by an appointment with a test and a discharge

**Fig. 6:**
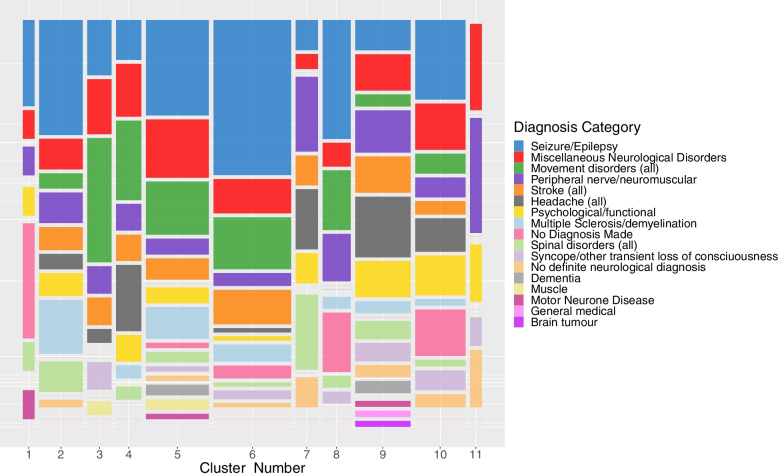
Mosaic plot showing the proportion of patients from each diagnosis category falling into each of the 11 clusters. Note that the width of the vertical columns in the plot represent the relative size of the clusters. Diagnoses with significant results are: Seizure/epilepsy, Movement disorders, Headache, and ‘No diagnosis made’

Analysis of the characteristics of the patients falling into each cluster reveals that there is no evidence that cluster membership is dependent on sex, age at first appointment, or time to referral. However, there is some evidence that cluster membership is dependent on diagnosis category. Analysis of the individual diagnosis categories shows that there are 3 diagnoses which differ significantly within the clusters, these are seizure/epilepsy, movement disorders, and headache (see Table 4). Visualisation of the diagnosis categories within the clusters reveals further patterns (Fig. 6).

**Table 4:** Patient characteristics for all 11 clusters

	1 (*n* = 12)	2 (*n* = 45)	3 (*n* = 25)	4 (*n* = 26)	5 (*n* = 65)	6 (*n* = 80)	7 (*n* = 23)	8 (*n* = 29)	9 (*n* = 57)	10 (*n* = 52)	11 (*n* = 12)	*p-value*
Sex (%):												
Female	7 (58)	22 (49)	10 (40)	9 (35)	36 (55)	31 (39)	14 (61)	15 (52)	36 (63)	25 (48)	6 (50)	*0.186*
Male	5 (42)	23 (51)	15 (60)	17 (65)	29 (45)	49 (61)	9 (39)	14 (48)	21 (37)	27 (52)	6 (50)	
Mean age at first appointment (SD)	43.7 (15.9)	51.9 (17.7)	52.0 (21.9)	54.6 (20.8)	48.8 (18.6)	48.9 (18.4)	54.7 (17.3)	44.4 (20.9)	51.8 (17.9)	40.4 (16.7)	53.4 (15.4)	*0.079*
Time from referral in weeks (SD)	11.5 (9.9)	11.9 (8.8)	12.3 (8.3)	15.5 (9.9)	14.5 (9.1)	13.9 (9.2)	13.8 (8.6)	16.1 (8.5)	9.8 (8.6)	10.7 (7.2)	14.7 (6.6)	*0.204*
Diagnosis Category (%):												
Seizure/epilepsy	3 (25.0)	15 (33.3)	4 (16.0)	3 (11.5)	18 (27.7)	36 (45.0)	2 (8.7)	10 (34.5)	5 (8.8)	12 (23.1)	-	***0.002***
Miscellaneous Neurological Disorders	1 (8.3)	4 (8.9)	4 (16.0)	4 (15.4)	11 (16.9)	8 (10.0)	1 (4.3)	2 (6.9)	6 (10.5)	7 (13.5)	3 (25.0)	*0.797*
Movement Disorders	-	2 (4.4)	9 (36.0)	6 (23.1)	10 (15.4)	12 (15.0)	-	5 (17.2)	2 (3.5)	3 (5.8)	-	***0.003***
Peripheral nerve/neuromuscular	1 (8.3)	4 (8.9)	2 (8.0)	2 (7.7)	3 (4.6)	3 (3.8)	5 (21.7)	4 (13.8)	7 (12.3)	3 (5.8)	4 (33.3)	*0.060*
Stroke	-	3 (6.7)	2 (8.0)	2 (7.7)	4 (6.2)	8 (10.0)	2 (8.7)	-	6 (10.5)	2 (3.8)	-	*0.720*
Headache	-	2 (4.4)	1 (4.0)	5 (19.2)	-	1 (1.3)	4 (17.4)	-	10 (17.5)	5 (9.6)	-	***0.001***
Psychological/functional	1 (8.3)	3 (6.7)	-	2 (7.7)	3 (4.6)	1 (1.3)	2 (8.7)	-	6 (10.5)	6 (11.5)	2 (16.7)	*0.154*
Multiple Sclerosis/demyelination	-	7 (15.6)	-	1 (3.8)	6 (9.2)	4 (5.0)	-	1 (3.4)	2 (3.5)	1 (1.9)	-	*0.079*
No Diagnosis Made	4 (33.3)	-	-	-	1 (1.5)	3 (3.8)	-	5 (17.2)	-	7 (13.5)	-	***0.0005***
Spinal disorders	1 (8.3)	4 (8.9)	-	1 (3.8)	2 (3.1)	1 (1.3)	5 (21.7)	1 (3.4)	3 (5.3)	1 (1.9)	-	*0.020*
Syncope/transient loss of consciousness	-	-	2 (8.0)	-	1 (1.5)	2 (2.5)	-	1 (3.4)	3 (5.3)	3 (5.8)	1 (8.3)	*0.499*
No definite neurological diagnosis	-	1 (2.2)	-	-	1 (1.5)	1 (1.3)	2 (8.7)	-	2 (3.5)	2 (3.8)	2 (25.0)	*0.095*
Dementia	-	-	-	-	2 (3.1)	-	-	-	2 (3.5)	-	-	*0.374*
Muscle	-	-	1 (4.0)	-	2 (3.1)	-	-	-	-	-	-	*0.278*
Motor Neurone Disease	1 (8.3)	-	-	-	1 (1.5)	-	-	-	1 (1.8)	-	-	*0.207*
Brain tumour	-	-	-	-	-	-	-	-	1 (1.8)	-	-	*0.655*
General medical	-	-	-	-	-	-	-	-	1 (1.8)	-	-	*0.641*

After adjusting for multiple testing statistically significant *p*-values are highlighted in bold

Figure 6 shows that a large proportion of seizure/epilepsy patients fall into cluster 6, the cluster with longer sequences of a standard appointment types, some of whom have tests ordered at their first appointment. Patients with headache disorders fall largely into clusters 4,7,9, all of which are clusters with high rates of discharge. This indicates that those patients with headache disorders who aren’t discharged at their first appointment (see Table 1), are likely to be discharged at their second appointment. Patients with movement disorders are more likely to fall into cluster 3, with moderate proportions in cluster 4,5, 6 and 8. Cluster 3 contains the patients with the longest sequences and therefore the highest number of appointments in the 18-month period.

## Discussion

There has been very little previous work to examine the types of appointments, and the sequence in which they occur, within outpatient neurology departments. This study helps to fill a gap in current understanding and provides a basis on which future work can be built. It is a starting point for understanding the current situation and provides evidence for the types of change that may be needed.

Many patients attended only one appointment and were not offered any follow-up within the neurology outpatient clinic. Within this group of patients there are a number of diagnostic categories where it is likely that a patient has been referred on to a different service after their first and only appointment; for example, patients with brain tumour referred to neuro-oncology; those with psychological and functional disorders referred on to relevant services including neuropsychology or neuropsychiatry; and those with ‘general medical’ diagnoses referred to different services.

Using SSA to explore neurology appointments has shown that there are many and varied ways that patients interact with neurology outpatient services. There is, in essence, no ‘one-size-fits-all’ pattern, even within single diagnostic categories. However, some patterns of similarities can be seen. We found that many patients return for follow-up on an underlying six-monthly cycle. The present study cannot tell us what drives this, but there are several possible explanations such as scheduling based on traditional outpatient pathways, patient behaviour and expectations, and administrative factors. Results in the present study show a ‘decay’ of the six-monthly cycle suggesting that variation in appointment scheduling emerges over the duration of patient follow up. This could be due to condition-specific differences (for example the timing of particular diagnostic investigations or treatments) or patient-specific differences (for example, patient expectations, or the level of support required) in appointment scheduling. This analysis helps to illustrate these varying patterns and the need for service planning to accommodate a wide range of scheduling patterns.

We found eleven distinct clusters of sequence types which describe within them broadly similar patterns of appointment sequence. Within these clusters there are some patterns common to particular diagnostic categories. For example, those with headache disorders are often discharged at the first appointment. By contrast, patients with movement disorders are seen for regular follow-up appointments. It is likely that such differences reflect condition-specific requirements for ongoing specialist clinic management. Primary headache disorders can often be managed in primary care, although some patients require neurologist input to guide primary care management [24]. Other chronic neurological conditions such as Parkinson’s disease (PD) are likely to require ongoing neurologist supervision due to the specialist nature of disease management, and National Institute for Health and Care Excellence (NICE) guidelines for PD suggest follow up appointments should be scheduled every 6 to 12 months [25], which may go some way to accounting for the 6 monthly cycle seen in Fig. 4.

Managing the number, frequency and type of individual patient follow-up appointments relies on many different factors, including the type of diagnosis given. This study shows that there are varied ways in which patients interact with neurology services, and although there are some commonalities between patients with the same diagnosis, there are also differences. This indicates the need for a flexible approach to appointment planning, a conclusion which is supported by the Getting It Right First Time (GIRFT) report released in September 2021. This report recommends that, for all patients with chronic neurological conditions, outpatient departments should “consider arranging clinically triggered follow-ups for patients with pending results, personalised patient-initiated follow-ups for patients with disease in remission or with stable disease, as well as the traditional timed follow-up appointments” [5].

This study has also shown that some patients have unexpected sequences, for example being discharged on a first appointment and yet returning for further follow-up. It is likely that a number of factors are responsible for this, such as an initial discharge being conditional upon the outcome of diagnostic investigations, a further appointment requested by the patient’s GP to explain investigation results where the GP may lack capacity to relay such information, or a patient initiating a follow-up appointment through contact with a different member of the booking team. This finding needs further investigation to explore the extent to which the observed appointment sequences deviate from planned sequences, in order to better understand what drives such differences.

### Limitations

This study focuses on a single clinic, so it is limited by both the amount of data available and the generalisability of the findings. Even with data from multiple clinics different types of analysis would need to be undertaken to move from the observational analysis of SSA to an understanding of why we see the results we have found. Furthermore, there are many stages during SSA where a different decision, for example to use a different algorithm to measure dissimilarity, could have effects on the results. More work needs to be done to understand the magnitude of the effects of choosing different parameters.

In this study we analyse the results at the level of diagnostic category, even though for some individuals more specific diagnosis will have been made. This is necessary both because the size of the study limits the amount of detail we can observe, and because specific diagnosis was not routinely coded at neurology outpatient appointments when the data for this study was collected. Routine coding of diagnoses, coupled with larger datasets would allow for a more detailed analysis of the differences in appointment sequences between diagnoses.

### Benefits

We have shown that neurology outpatients is a complex environment. Patients have many different diagnoses, with significant variation in multiple dimensions, including patient needs and expectations, as well as a multitude of condition-specific and clinician-directed elements, all of which influence the planned and/or observed number and types of appointments. Using SSA has allowed us to visualise distinct sequences and see which types of sequences are most common. Identifying common patterns, whilst acknowledging the breadth of the differences, can help to inform future planning. This study provides a starting point for understanding neurology outpatients and should offer support in the wider effort to meet targets and standards, and ultimately to improve patient access based on clinical need, as well care delivery.

### Future work

The data for this study were acquired prior to the COVID-19 pandemic, and it is possible that data collected post-pandemic would show different patterns in appointment sequences. For example, the use of remote services adopted during the pandemic, such as telehealth consultations instead of face-to-face appointments, might have changed the pattern and timing of follow-up appointments. Further work is needed to verify this. In addition, it is possible that the release of the GIRFT report in 2021 may have influenced the policy and practice of when and how follow-up appointments are offered. Examining the impact of these two changes offers an interesting avenue for future research.

Future work could also expand the current research to a national level which would facilitate much more comprehensive understanding of patterns of outpatient care. With larger datasets more detail could be examined, for example looking in more depth at diagnosis-specific sequences. In addition, national level research could also be used to highlight differences within and between regions, examining variation and its potential causes. This study only looks at a single clinic in a region which has a particularly low consultant to population ratio; it would be informative, for example, to compare this to the types of appointment sequences seen in areas which are better resourced with consultant neurologists.

## Conclusion

SSA is a useful methodology for exploring patterns of outpatient appointments, especially patterns of appointment type. Neurology outpatient appointments show great diversity across all diagnostic categories, but there are some patterns which are more common within specific diagnoses. Information about these common patterns could be used to inform the structure of future outpatient appointments, especially when considering initiatives such as the NHS Outpatient Transformation Program.

### Supplementary Information

**Additional file 1: Supplemental Table 1.** Patient characteristics for the 5 clusters resulting from SSA on the sequences focussed on appointment timing. Chi-square tests were carried out to test for significance and statistically significant *p*-values are highlighted in bold (Bonferroni adjustments for multiple testing were used). **Supplemental Figure 1.** Visualisation of the 5 clusters resulting from SSA on the sequences focussed on appointment timing. The most common sequence in each cluster is oriented at the base of the y-axis and the height of the bars represents the frequency of that sequence within the cluster. Cluster size is included in brackets in the y-axis title.

## Data Availability

The datasets analysed during this study are not publicly available due to patient data confidentiality and restrictions imposed by the Health Research Authority (HRA). Data are available from the authors on reasonable request and with permission of the HRA.

## Abbreviations

DMT: Disease Modifying Therapy
GIRFT: Getting It Right First Time
GP: General Practitioner
MS: Multiple Sclerosis
NHS: National Health Service
NICE: National Institute for Health and Care Excellence
PD: Parkinson’s Disease
SSA: State Sequences Analysis
