# Supplementary material for: Discovering patterns in outpatient neurology appointments using state sequence analysis
Source: BMC Health Serv Res. 2023 Nov 6;23:1208. doi: 10.1186/s12913-023-10218-y (PMC10626691; doi:10.1186/s12913-023-10218-y)
Supplement: Supplementary file 1 — Additional file 1: Supplemental Table 1. Patient characteristics for the 5 clusters resulting from SSA on the sequences focussed on appointment timing. Chi-square tests were carried out to test for significance and statistically significant p-values are highlighted in bold (Bonferroni adjustments for multiple testing were used). Supplemental Figure 1. Visualisation of the 5 clusters resulting from SSA on the sequences focussed on appointment timing. The most common sequence in each cluster is oriented at the base of the y-axis and the height of the bars represents the frequency of that sequence within the cluster. Cluster size is included in brackets in the y-axis title. [file 12913_2023_10218_MOESM1_ESM.docx]

**Supplemental materials**

|  | 1  (n=163) | 2  (n=49) | 3  (n=89) | 4  (n=71) | 5  (n=54) | *p-value* |
| --- | --- | --- | --- | --- | --- | --- |
| Sex (%): |  |  |  |  |  |  |
| Female | 86 (53) | 22 (45) | 37 (42) | 37 (52) | 29 (54) | *0.42* |
| Male | 77 (47) | 27 (55) | 52 (58) | 34 (48) | 25 (46) |  |
|  |  |  |  |  |  |  |
| Mean age at first appointment (SD) | 47.1 (18.9) | 50.9 (16.9) | 52.4 (19.7) | 50.6 (17.2) | 46.3 (19.4) | *0.31* |
|  |  |  |  |  |  |  |
| Time from referral in weeks (SD) | 12.2 (9.1) | 14.7 (9.3) | 13.9 (8.7) | 12.7 (8.5) | 12.3 (8.3) | *0.85* |
| Diagnosis Category (%): |  |  |  |  |  |  |
| Seizure/epilepsy | 45 (27.6) | 12 (24.5) | 15 (16.9) | 22 (31.0) | 14 (25.9) | *0.441* |
| Miscellaneous Neurological Disorders | 18 (11.0) | 4 (8.2) | 12 (13.5) | 8 (11.3) | 9 (17.7) | *0.820* |
| Movement Disorders | 15 (9.2) | 14 (28.6) | 11 (12.4) | 6 (8.5) | 3 (5.5) | ***0.003*** |
| Peripheral nerve/neuromuscular | 14 (8.6) | 1 (2.1) | 15 (16.9) | 6 (8.5) | 2 (3.7) | *0.041* |
| Stroke | 12 (7.4) | 3 (6.1) | 6 (6.7) | 6 (8.5) | 2 (3.7) | *0.888* |
| Headache | 12 (7.4) | 4 (8.2) | 5 (5.6) | 4 (5.6) | 3 (5.5) | *0.955* |
| Psychological/functional | 10 (6.1) | 2 (4.1) | 4 (4.5) | 6 (8.5) | 4 (7.4) | *0.865* |
| Multiple Sclerosis/demyelination | 6 (3.7) | 4 (8.2) | 3 (3.4) | 6 (8.5) | 3 (5.5) | *0.446* |
| No Diagnosis Made | 10 (6.1) | - | 6 (6.7) | 2 (2.8) | 2 (3.7) | *0.355* |
| Spinal disorders | 7 (4.3) | 1 (2.1) | 5 (5.6) | 4 (5.6) | 2 (3.7) | *0.912* |
| Syncope/transient loss of consciousness | 5 (3.1) | 1 (2.1) | 2 (2.2) | - | 5 (9.3) | *0.051* |
| No definite neurological diagnosis | 4 (2.5) | 3 (6.1) | 1 (1.1) | 1 (1.4) | 2 (3.7) | *0.399* |
| Dementia | 1 (0.6) | - | 1 (1.1) | - | 2 (3.7) | *0.234* |
| Muscle | 1 (0.6) | - | 1 (1.1) | - | 1 (1.9) | *0.919* |
| Motor Neurone Disease | 2 (1.2) | - | 1 (1.1) | - | - | *0.870* |
| Brain tumour | 1 (0.6) | - | - | - | - | *0.803* |
| General medical | - | - | 1 (1.1) | - | - | *0.641* |

**Supplemental Table 1:** Patient characteristics for the 5 clusters resulting from SSA on the sequences focussed on appointment timing. Chi-square tests were carried out to test for significance and statistically significant p-values are highlighted in bold (Bonferroni adjustments for multiple testing were used).

**Supplemental Figure 1:** Visualisation of the 5 clusters resulting from SSA on the sequences focussed on appointment timing. The most common sequence in each cluster is oriented at the base of the y-axis and the height of the bars represents the frequency of that sequence within the cluster. Cluster size is included in brackets in the y-axis title.
